# Intra‐ and inter‐fractional variations of tumors with fiducial markers measured using respiratory‐correlated computed tomography images for respiratory gated lung stereotactic body radiation therapy

**DOI:** 10.1002/acm2.14280

**Published:** 2024-01-22

**Authors:** Yuki Manabe, Takehiro Shiinoki, Koya Fujimoto, Kazushi Ueda, Masako Karita, Taiki Ono, Miki Kajima, Hidekazu Tanaka

**Affiliations:** ^1^ Department of Radiation Oncology Yamaguchi University Graduate School of Medicine Ube Yamaguchi Japan

**Keywords:** fiducial marker, lung tumor, stereotactic body radiation therapy

## Abstract

**Purpose:**

This study evaluated the intra‐ and inter‐fractional variation of tumors with fiducial markers (FMs), relative to the tumor‐FM distance, to establish how close an FM should be inserted for respiratory‐gated stereotactic body radiation therapy (RG‐SBRT).

**Methods:**

Forty‐five lung tumors treated with RG‐SBRT were enrolled. End‐expiratory computed tomography (CT) (CT_plan_) and four‐dimensional‐CT (4D‐CT) scans were obtained for planning. End‐expiratory CT (CT_fr_) scanning was performed before each fraction. The FMs were divided into two groups based on the median tumor‐FM distance in the CT_plan_ (D_p_). For the intra‐fractional variation, the correlations between the corresponding tumor and FM intra‐fractional motions, defined as the centroid coordinates of those in each 0–90% phase, with the 50% phase of 4D‐CT as the origin, were calculated in the left‐right, anterior‐posterior, and superior‐inferior directions. Furthermore, the maximum difference in the tumor‐FM distance in each phase of 4D‐CT scan, based on those in the 50% phase of 4D‐CT scan (D_max_), was obtained. Inter‐fractional variation was defined as the maximum distance between the tumors in CT_plan_ and CT_fr_, when the CT scans were fused based on each FM or vertebra.

**Results:**

The median D_p_ was 26.1 mm. While FM intra‐fractional motions were significantly and strongly correlated with the tumor intra‐fractional motions in only anterior‐posterior and superior‐inferior directions for the D_p_ > 26 mm group, they were significantly and strongly correlated in all directions for the D_p_ ≤ 26 mm group. In all directions, D_max_ values of the D_p_ ≤ 26 mm group were lower than those of the D_p_ > 26 mm group. The inter‐fractional variations based on the D_p_ ≤ 26 mm were smaller than those on the D_p_ > 26 mm and on the vertebra in all directions.

**Conclusions:**

Regarding intra‐ and inter‐fractional variation, FMs for D_p_ ≤ 26 mm can increase the accuracy for RG‐SBRT.

## 1 INTRODUCTION

Stereotactic body radiation therapy (SBRT) delivers a precise high dose of radiation to a limited target volume. Owing to the good local control achieved using this technique, SBRT is widely performed for inoperable early‐stage primary and metastatic lung cancer.^1^, ^2^, ^3^, ^4^, ^5^, ^6^

Lung tumors can move by more than 30 mm.^7^ In lung SBRT, the tumor motion during breathing results in significant geometric uncertainty during high‐dose delivery to the target. This geometric uncertainty can be addressed by the introduction of an expanded internal target volume (ITV). However, a large target volume can increase the irradiation dose to the surrounding normal tissue and increase the risk of adverse events such as radiation pneumonitis.^8^, ^9^, ^10^ To reduce geometric uncertainty and adverse event risk, SBRT for lung tumors with severe respiratory motion requires appropriate motion management.^11^ Several respiratory motion management methods have been used, including breath holding, respiratory gating, and dynamic tumor tracking.^12^, ^13^, ^14^ In these respiratory motion management methods, fiducial markers (FMs), inserted either in the tumor itself or in close proximity, are often used as internal surrogates to localize the tumor.^15^, ^16^

At our institution, respiratory‐gated SBRT (RG‐SBRT) for lung tumors is performed using the real‐time tumor monitoring system.^17^, ^18^ This method can be used to monitor FM during irradiation using two fluoroscopic images. The treatment beam to the target was turned on only when the selected FM was located within a few millimeters of the planned three‐dimensional position of the FM. RG‐SBRT is effective in reducing ITV. To achieve highly precise RG‐SBRT using a real‐time tumor monitoring system, the positional accuracy of the FM as an internal surrogate for locating the tumor is important.

Several authors have reported the intra‐fractional variation between the FM used as an internal surrogate and the lung tumor during respiration.^19^, ^20^ Inter‐fractional variations of lung tumors with respect to FM due to various factors, such as tumor distortion by daily radiation therapy and FM migration, have also been reported.^20^, ^21^ In lung RG‐SBRT, the inter‐fractional variation as well as the intra‐fractional variation of the lung tumor can result in geometric uncertainty in dose delivery to the tumor, increasing the target volume and the risk of adverse events if an inappropriate FM is used as an internal surrogate.

An FM is often implanted using an endobronchial approach because of its minimally invasive nature and short intervention time.^16^, ^22^ In this approach, the locations where the FMs can be placed are limited because the FMs are implanted along the small bronchi. Physicians try to place the FM transbronchially close to the lung tumor; however, it is difficult to place the FMs within a few millimeters of the tumor. A few studies have shown that the intra‐ or inter‐fractional variation of lung tumors correlates with the distance between the lung tumor and FM.^23^, ^24^ However, little is known about how closely the FM needs to be inserted into lung tumors during SBRT.

Herein, we aimed to quantitatively evaluate the intra‐ and inter‐fractional variations in lung tumors with FMs and compared the intra‐ and inter‐fractional variations based on the distance between the lung tumor and FM.

## 2 METHODS

### 2.1 Patients

Forty‐three patients treated for one–two lung tumors with RG‐SBRT in the end‐expiratory (EE) phase during free‐breathing using a real‐time tumor monitoring system between April 2017 and August 2021 were enrolled in this study. Patients requiring home oxygen therapy at the time of treatment were excluded. Patient characteristics are presented in Table 1.

**TABLE 1:** Characteristics of patients and tumors.

Characteristics	
Sex	
Male	30
Female	13
Age (years)	
Median [range]	80 [50−91]
Number of tumors	
1	41
2	2
Tumor volume (cc)	
Median [range]	2.1 [0.1−31.7]
Tumor location	
Right upper lobe	10
Right middle lobe	3
Right lower lobe	16
Left upper lobe	9
Left lower lobe	7
Number of CT_fr_s	
3	8
4	26
5	3
6	0
7	2
8	6
Number of inserted FMs	
2	1
3	15
4	26
5	2
6	1
Number of residual FMs in CT_plan_	
1	1
2	6
3	15
4	22
5	1

Abbreviations: CT_fr_, computed tomography scans obtained at the end‐expiratory phase before each fraction to confirm FM migration; FM, fiducial marker.

Prior to computed tomography (CT) simulation, 2−6 FMs were inserted near each tumor using bronchoscopy. The gold marker with diameter of 1.5 mm (Disposable Gold Marker; Olympus Medical Systems, Tokyo, Japan) was used as the FM. The FMs that dropped out before the CT simulation were excluded from the evaluation. Residual FMs were evaluated at the time of the CT simulation.

The Institutional Review Board approved this study and the requirement for written informed consent was waived because of its retrospective design.

### 2.2 CT data acquisition and contouring

A CT scanner (SOMATOM Definition AS; Siemens Healthcare, Erlangen, Germany) was used for CT imaging. One to two weeks after FM placement (median,7 days), CT simulations were performed for treatment planning, and each patient was immobilized in the supine position using a Vac‐Lok system (CIVCO Medical Solutions, Coralville, Iowa, USA). Four‐dimensional‐CT (4D‐CT) under free‐breathing and breath‐hold CT in the EE phase (CT_plan_) scans were obtained with a 2 mm slice thickness for planning. The CT scans were acquired while the respiratory phase was monitored using a real‐time position‐management system (Varian Medical Systems, Palo Alto, California, USA).

Breath‐hold CT in the EE phase (CT_fr_) was also obtained with a 2‐mm slice thickness before each fraction to confirm FM migration. If cone‐beam CT (CBCT) scanning was substituted to confirm FM migration, it was not included in the CT_fr_.

All CT image sets were imported into Eclipse treatment planning system (Varian Medical Systems, Palo Alto, California, USA). All FMs and lung tumors in 10 phases of 4D‐CT and CT_plan_ and lung tumors in each CT_fr_ sets were contoured with the treatment planning system by a radiation oncologist with 8 years of experience.

The centroid coordinates of the contoured FMs and lung tumors were acquired from each CT image. The three‐dimensional distance between the centroid of the tumor and that of the FM in CT_plan_ (D_p_) was calculated for each FM. The FMs were divided into two groups based on the median D_p_. When D_p_ was greater than 95^th^ percentile of D_p_, the FMs were excluded from the D_p_ groups.

### 2.3 Evaluation of the intra‐fractional variation using 4D‐CT

**FIGURE 1:**
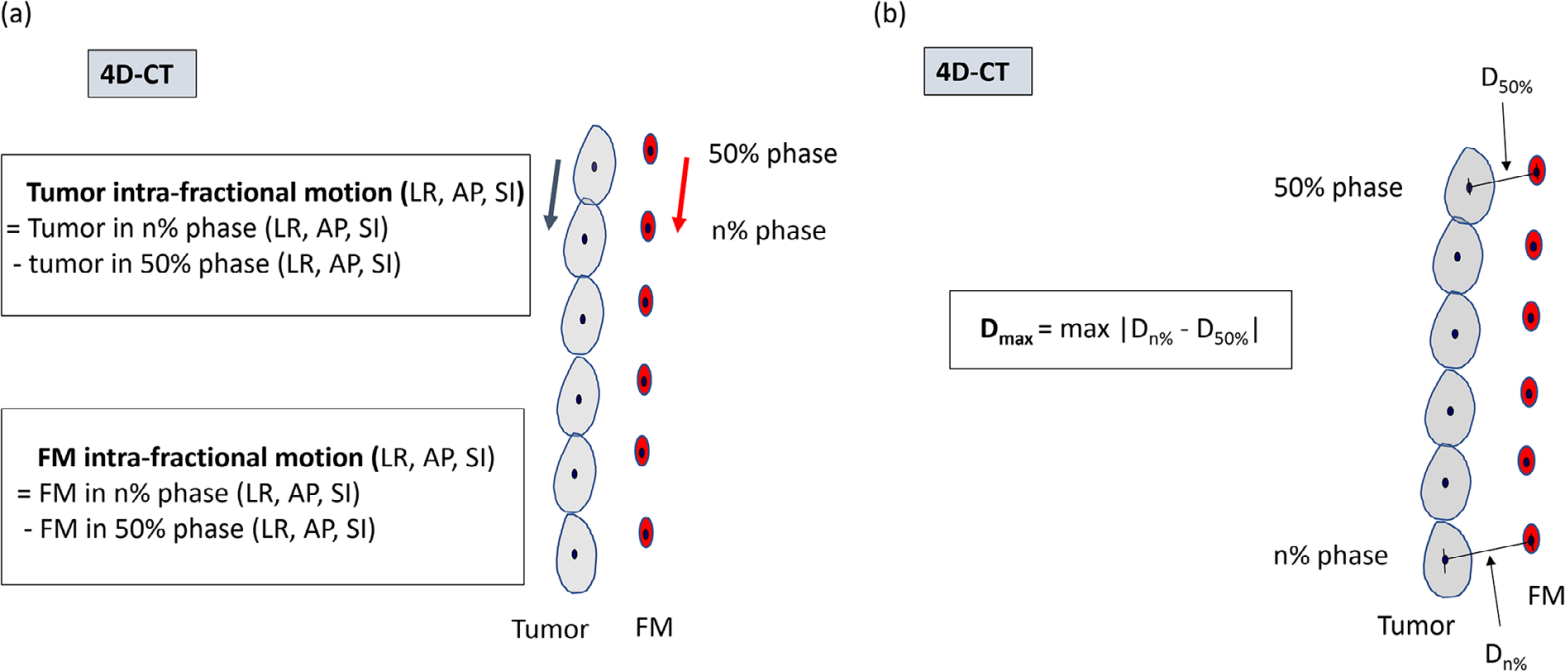
Definition of the tumor and fiducial marker (FM) intra‐fractional motions (a), and D_max_ for the evaluation of the intra‐fractional variation (b). Two items were considered in the evaluation of intra‐fractional variation using 4D‐CT scans: the correlation between tumor and FM intra‐fractional motions, and the difference between tumor and FM distance in each phase. (a) Tumor and FM intra‐fractional motions were defined as the centroid coordinates of the tumor and FM in each 0−90% phase, with the 50% phase of the 4D‐CT scanning used as the origin. (b) D_max_ is defined as the maximum difference in the distance between the tumor and FM in each phase of 4D‐CT scanning based on the distance between the tumor and FM in the 50% phase of 4D‐CT scanning.

In this study, the intra‐fractional variation was defined as the intra‐fractional motion of the tumor relative to the FM during respiration. 4D‐CT scans were used to assess the intra‐fractional variation. Two items were evaluated for intra‐fractional variation: the correlation between tumor and FM intra‐fractional motions and the difference between tumor and FM distance in each phase of 4D‐CT scanning (Figure 1).

First, the centroid coordinates of the tumor and the FM in each 0−90% phase with the 50% phase of the 4D‐CT scan as the origin were calculated as the tumor and the FM intra‐fractional motion. The correlation between the corresponding tumor and FM intra‐fractional motion was determined in the left‐right (LR), anterior‐posterior (AP), and superior‐inferior (SI) directions for each FM group based on the median D_p_.

Next, the distance (D_n%_; 0 ≤ *n* ≤ 90) between centroids of the tumor and FM was acquired in each 0−90% phase of 4D‐CT scan in the LR, AP, and SI directions. For each FM, the maximum difference (D_max_) between D_n%_ and D_50%_ was calculated in each direction. The D_max_ was compared between the two FM groups based on the median D_p_.

### 2.4 Evaluation of the inter‐fractional variation using breath‐hold CT at EE

CT_plan_ and CT_fr_ scans were used to assess the inter‐fractional variation. First, CT_fr_ scans were registered in CT_plan_ based on the vertebra. The distances between the centroids of the tumors on CT_plan_ and CT_fr_ scans were calculated in the LR, AP, and SI directions. In each tumor, the maximum distances among all CT_fr_‐based datasets were calculated as inter‐fractional variations based on the vertebra.

**FIGURE 2:**
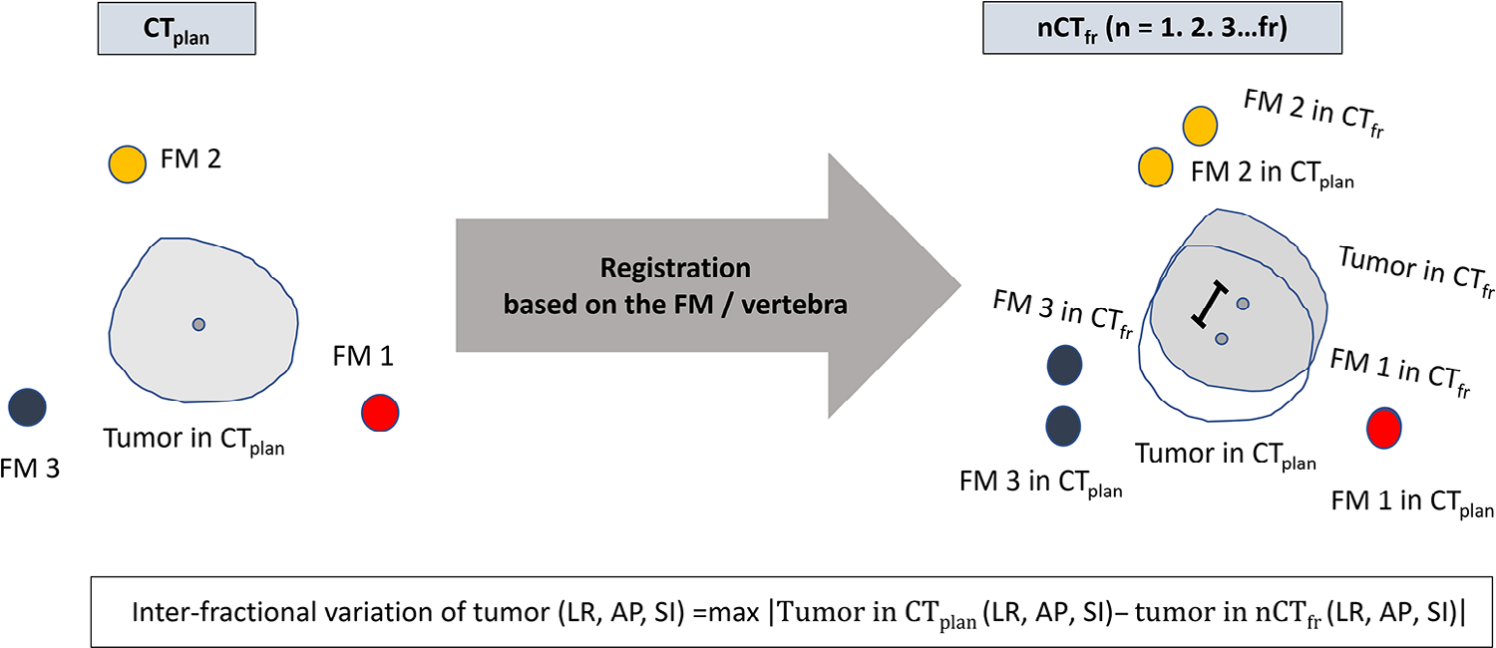
Definition of the inter‐fractional variation. Inter‐fractional variation was defined as the maximum distance between the centroid of the tumors captured on CT images at the end‐expiratory phase for treatment planning (CT_plan_) and before each fraction (CT_fr_) among CT_fr_‐based datasets when those CT scans were registered based on each fiducial marker (FM) or vertebra.

Next, CT_fr_ scans were registered in CT_plan_ based on each FM. The distances between the centroids of the tumors in CT_plan_ and CT_fr_ scans registered based on the FM were calculated in each direction. In each FM, the maximum distances among all CT_fr_‐based datasets were calculated as the inter‐fractional variations based on the FM (Figure 2).

The inter‐fractional variation values in each direction were compared between the three groups based on the two D_p_ groups and the vertebra group. If the FM had migrated during the treatment period, it was evaluated up to that point.

### 2.5 Analysis

All statistical analyses were performed using JMP ver16.1.0 (SAS Institute Inc., Cary, North Carolina, USA). Pearson's correlation coefficient was used to evaluate the correlation between the tumor and the FM intra‐fractional motion. A strong correlation was defined as a Pearson's |*R*| > 0.70. The Wilcoxon rank‐sum test was used to compare D_max_ values between the two D_p_ groups.^25^ To compare inter‐fractional variation between the three groups, based on the two D_p_ groups and the vertebral group, the Kruskal–Wallis test with Steel–Dwass test was used for analysis. Statistical significance was set at *p*‐values of < 0.05.

## 3 RESULTS

### 3.1 Inserting and grouping the FMs

Overall, 167 FMs were inserted near lung tumors using the endobronchial approach. One patient experienced pneumothorax as a side effect of FM insertion, but recovered on their own. Further, 16 FMs dropped out before the CT simulation.

Table 2 presents the characteristics of patients in the FM group. The median (range and 95^th^ percentile) value of the D_p_ was 26.1 mm (4.8−67.0  and 58.4 mm). Seven FMs were excluded because those D_p_ values were larger than the 95^th^ percentile of D_p_ (i.e., larger than 58.4 mm). Seventy‐four FMs were divided into the D_p_ ≤ 26 mm group and D_p_ > 26 mm group (Table 2).

**TABLE 2:** Characteristics of FM groups.

FM groups	D_p_ ≤ 26 mm (*N* = 74)	D_p_ > 26 mm (*N* = 70)
D_p_ (mm)		
Median [range]	18.7 [4.8−25.6]	34.1 [26.1−58.0]
Mean [SD]	17.5 [5.5]	36.3 [8.2]
Tumor location corresponding to the FM		
Right upper lobe	15	19
Right middle lobe	3	8
Right lower lobe	31	19
Left upper lobe	14	13
Left lower lobe	11	11

Abbreviations: D_p_, distance between the centroid of the tumor and that of the FM on computed tomography scans obtained at the end‐expiratory phase for treatment planning (CT_plan_);FM, fiducial marker; SD, standard deviation.

### 3.2 Intra‐fractional variation

**FIGURE 3:**
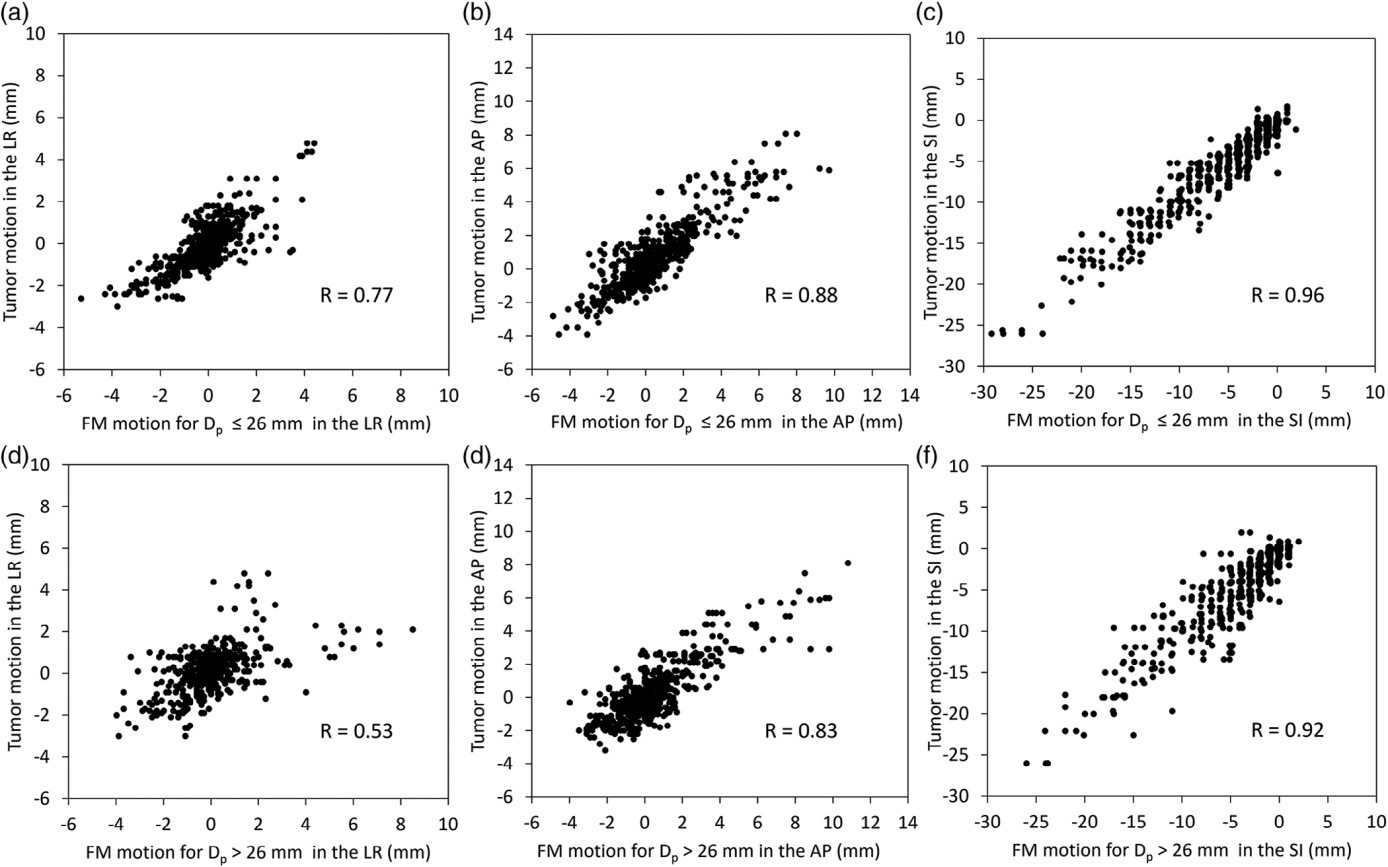
Scatter plot and Pearson's correlation coefficients between the corresponding tumor and fiducial marker (FM) motions defined as the centroid coordinates of the tumor and FM in each 0−90% phase with the 50% phase of the 4D‐CT scan as the origin. Those (a) in the left‐right, (b) the anterior‐posterior, and (c) the superior‐inferior directions for the D_p_ ≤ 26 mm group. (d) Left‐right, (e) anterior‐posterior, and (f) superior‐inferior directions in the D_p_ > 26 mm group.

D_max_ values and correlation between tumor and FM intra‐fractional motions were evaluated. Figure 3 shows the scatter plot and Pearson's correlation coefficients between the corresponding tumor and FM intra‐fractional motions. For the D_p_ ≤ 26 mm group, the FM intra‐fractional motions were significantly and strongly correlated with the tumor intra‐fractional motions in the LR (*R* = 0.77, *p* < 0.001), AP (*R* = 0.88, *p* < 0.001), and SI (*R* = 0.96, *p* < 0.001) directions. The FM intra‐fractional motions were significantly and strongly correlated with the tumor intra‐fractional motions only in the AP (*R* = 0.83, *p* < 0.001) and SI (*R* = 0.92, *p* < 0.001) directions in the D_p_ > 26 mm group. In the LR direction, FM intra‐fractional motion was significantly and moderately correlated with tumor intra‐fractional motion in the D_p_ > 26 mm group (*R* = 0.53, *p* < 0.001).

**FIGURE 4:**
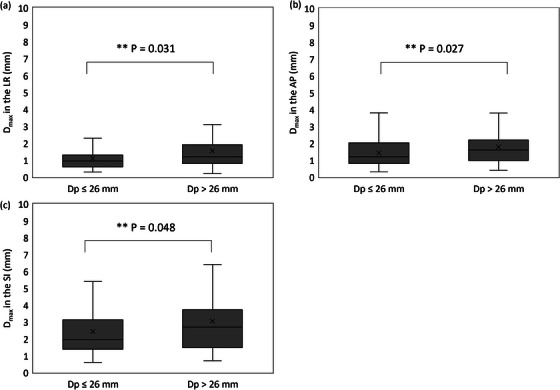
Maximum differences of the distance between tumor and fiducial marker (FM) (D_max_s) in the D_p_ ≤ 26 mm and in the D_p_ > 26 mm groups (a) in the left‐right, (b) the anterior‐posterior, and (c) the superior‐inferior directions. D_max_ was defined as the maximum difference in the distance between the tumor and the FM in each phase of 4D‐CT scanning, based on the distance between the tumor and the FM in the 50% phase of 4D‐CT scans.

Figure 4 shows the D_max_ values in the two D_p_ groups. The median (quartile) D_max_ values in the LR, AP, and SI directions were 1.0 mm (0.6−1.3), 1.2 mm (0.8−2.0), and 2.0 mm (1.4−3.1) in the D_p_ ≤ 26 mm group, and 1.2 mm (0.8−1.9), 1.6 mm (1.0−2.2), and 2.7 mm (1.5−3.7) in the D_p_ > 26 mm group, respectively. The D_max_ values in the D_p_ ≤ 26 mm group were smaller than those in the D_p_ > 26 mm group in the LR (*p* = 0.031), AP (*p* = 0.027), and SI (*p* = 0.048) directions.

### 3.3 Inter‐fractional variation

The median (quartile) inter‐fractional variation values in the LR, AP, and SI directions were 1.0 mm (0.5−1.6), 1.0 mm (0.7−1.6), and 1.4 mm (1.0−1.9), based on the FM in the D_p_ ≤ 26 mm group, 1.6 mm (1.1−2.5), 1.8 mm (1.3−2.8), and 1.9 mm (1.1−3.1), based on the FM in the D_p_ > 26 mm group, and 1.5 mm (0.9−2.6), 2.9 mm (1.4−4.7), and 4.0 mm (2.6−5.9), based on the vertebra, respectively.

The inter‐fractional variation values based on the FM in the D_p_ ≤ 26 mm group, based on the FM in the D_p_ > 26 mm group, and based on the vertebra were significantly different in the LR (*p* < 0.001), AP (*p* < 0.001) and SI (*p* < 0.001) directions by Kruskal–Wallis test.

**FIGURE 5:**
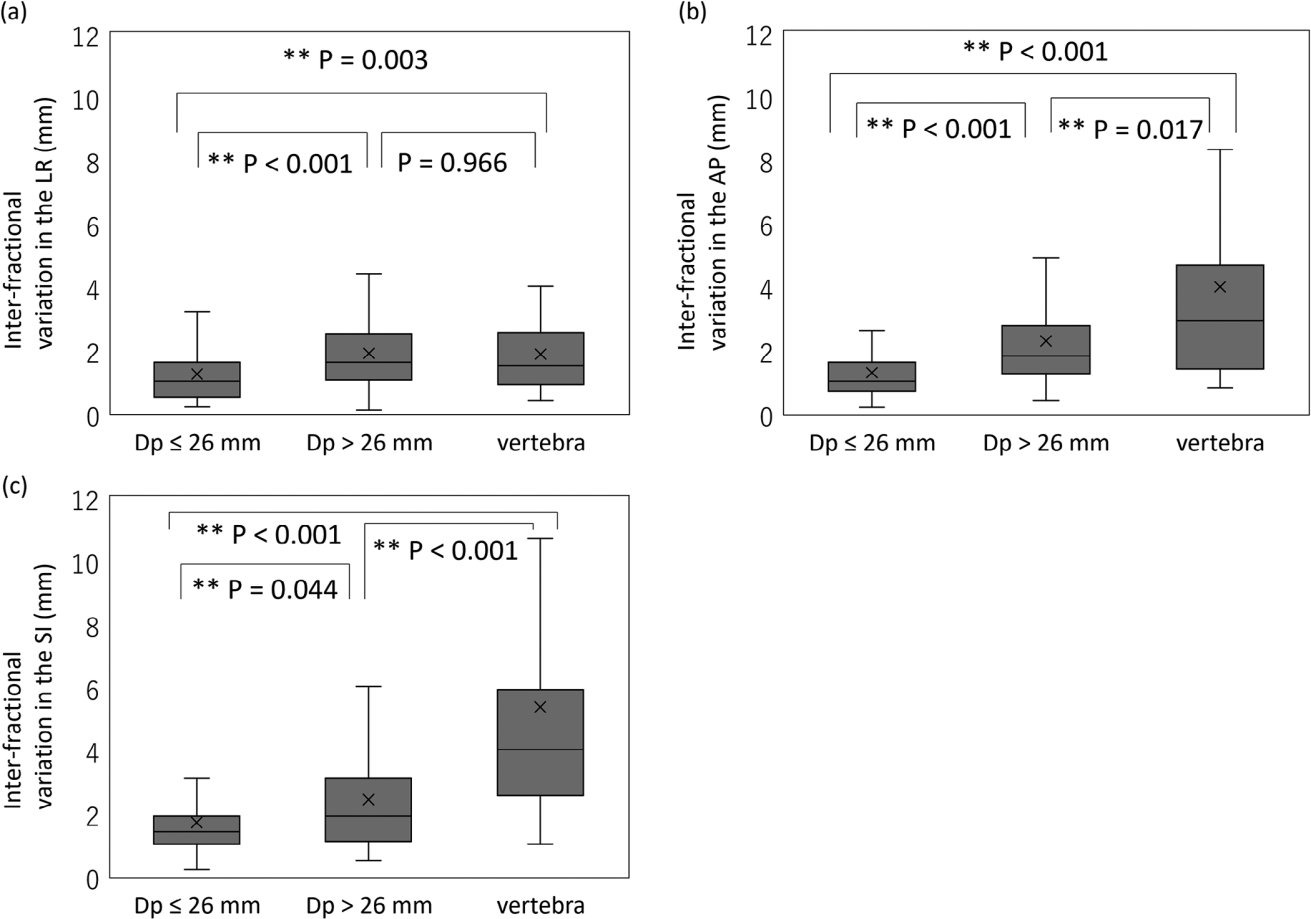
Differences of the inter‐fractional variations based on the D_p_ ≤ 26 mm group, on the D_p_ > 26 mm group, and on the vertebra (a) in the left‐right, (b) the anterior‐posterior, and (c) the superior‐inferior directions. Inter‐fractional variation was defined as the maximum distance between the centroid of the tumors in CT scans at end‐expiration for treatment planning (CT_plan_) and in CT scans at end‐expiration before each fraction (CT_fr_) among CT_fr_‐based datasets when the CT scans were fused based on each fiducial marker (FM) or vertebra.

The inter‐fractional variation values in the D_p_ ≤ 26 mm group were significantly smaller than those in the D_p_ > 26 mm group in the LR (*p* < 0.001), AP (*p* < 0.001), and SI (*p* = 0.044), respectively. The inter‐fractional variation values in the D_p_ ≤ 26 mm group were also significantly smaller than those in the vertebral group in the LR (*p* = 0.003), AP (*p* < 0.001), and SI (*p* < 0.001) directions. The inter‐fractional variations in the D_p_ > 26 mm group were significantly smaller than those in the vertebral group in the AP (P = 0.017) and SI (*p* < 0.001) directions. In the LR direction, the inter‐fractional variation was not significantly different between the D_p_ > 26 mm and vertebra groups (*p* = 0.966) (Figure 5).

Among the residual FMs at the time of CT_plan_ imaging, six FMs migrated or dropped out of the CT_fr_ scanning during the treatment period.

## 4 DISCUSSION

In this study, the FM was inserted using an endobronchial approach. As this method involves the placement of the FM in the bronchus, it may be difficult to place the FM near the tumor. Another common method of FM insertion is the transcutaneous approach. The transcutaneous approach has the potential to place FMs closer to the tumor compared to the endobronchial approach. However, the transcutaneous approach has a higher risk of pneumothorax as a complication of chest tube treatment.^26^, ^27^ Meanwhile, the endobronchial approach has a lower risk of pneumothorax.^16^, ^23^, ^27^, ^28^ In the present study, only one case of pneumothorax improved with follow‐up. The endobronchial approach is preferable for the minimally invasive placement of FMs.

Willmann et al.^29^ reported that tumor and FM motions correlated well in the AP and SI directions in their study using 4D‐CT scanning. Our results are comparable to these results. Furthermore, in our study, a correlation between tumor and FM intra‐fractional motion was observed in the LR direction. A correlation between tumor intra‐fractional motion and FM intra‐fractional motion was also observed in the D_p_ > 26 mm group, but the correlation was found to decrease, especially in the LR direction, in our study.

Only a few studies have indicated the specific distances between tumors and FMs.^25^, ^30^, ^31^ Akasaka et al.^25^ analyzed the three‐dimensional distance of the coiled FM to the lung tumor in CT scans for planning at the expiratory and inspiratory phases (D_diff_) and the ratio of ITV to gross tumor volume (GTV) (ITV/V_GTV_) created by 4D‐CT and CT scans at the inspiratory phase for treatment planning. They showed that D_diff_ and ITV/V_GTV_ were smaller when the distance between the edges of the tumor and the FM was less than 10 mm. Our study defined the distance between the tumor and FM as the distance between both centroids, while they defined the distance between both margins (i.e., closest distance). Therefore, regarding the difference between the 26 and 10 mm distances, it is necessary to consider the size of the tumor and the FM. In a preliminary study of FMs using 4D‐CT scans in eight patients, Yamasaki et al.^30^ reported that misalignment in the expiratory phase was significantly greater when the distance between the FM and the lung tumor was 30 mm or greater than when it was less than 30 mm. They also reported that the misalignment in the expiratory phase was smaller than 2.5 mm when the distance between the FM and lung tumor was less than 25 mm in their study. They concluded that the FM should be inserted within approximately 25 mm from the lung tumor. Smith et al.^31^ analyzed the motion correlation between lung tumors and the surrounding tissue in 10 lung cancer patients with deformable registration between the EE and end‐inspiratory phase on 4D‐CT scans. They suggested that an FM inserted within 30 mm of the lung tumor represented tumor motion with an error within 3 mm in many patients. These reports^25^, ^30^, ^31^ on intra‐fractional variation support our findings: D_max_ values of the FMs for D_p_ ≤ 26 mm were smaller than those of the FMs for D_p_ > 26 mm in all directions. Considering our results, the prior reports on intra‐fractional variation,^25^, ^30^, ^31^ and the distance from the tumor where the FM can be inserted into the bronchus, 26 mm may be a reasonable distance between the FM and the lung tumor.

Furthermore, the present study showed that the inter‐fractional variations in the D_p_ ≤ 26 mm group were also significantly smaller than those in the D_p_ > 26 mm group in all directions. As the inter‐fractional variation as well as the intra‐fractional variation of the lung tumor can affect the ITV, the FM for D_p_ ≤ 26 mm can be expected to reduce the ITV in terms of both intra‐ and inter‐fractional variations. Therefore, a tumor‐FM distance of 26 mm or less can be one of the distance criteria for the FM placement in terms of both intra‐ and inter‐fractional variation values of the lung tumor. No other studies have examined inter‐fractional variation with respect to the specific distance between the lung tumor and the FM. Roman et al.^24^ suggested that smaller distances between the lung tumor and FMs resulted in smaller inter‐fractional variation values in seven patients with locally advanced lung cancers. This report supports our study findings.

Image‐guided set‐up based on the FM is considered to have less positional variation than that based on the vertebra.^32^ The present study showed similar results. Furthermore, our simulated study using CT images showed that the inter‐fractional variation with a set‐up based on an FM was smaller than that with a set‐up based on the vertebra even when the distance between the tumor and FM was greater than 26 mm (D_p_ > 26 mm). According to our results, even if the FM is not implanted within 26 mm from the tumor, the FM has a certain efficacy in terms of the reduction of the inter‐fractional variation. However, in the present study, there was no significant difference in inter‐fractional variation in the LR direction between the set‐up based on the vertebra and FM in the D_p_ > 26 mm group. This finding may be due to the lower correlation in the LR direction than in other directions between the FM intra‐fractional motion of D_p_ > 26 mm and tumor intra‐fractional motion.

Our study has some limitations. Motion artifacts in respiratory‐correlated CT images can affect contouring. In addition, we used CT images with 2 mm slice thickness to evaluate the position of 1.5‐mm the diameter FM. The CT slice thickness can also affect contouring. Contouring uncertainty may lead to overestimation or underestimation of tumors and FMs. Furthermore, the poor reproducibility of breath‐holding with the use of expiratory breath‐holding CT scans in the assessment of inter‐fractional variation may have affected the results.^20^ This limitation can be improved using 4D‐CT or 4D‐CBCT scanning.^23^ However, a problem of increased radiation exposure exists.^33^

## 5 CONCLUSION

This study evaluated intra‐ and inter‐fractional variations based on the distance between lung tumors and FMs. A distance of 26 mm or less between the FM and lung tumors improved both intra‐ and inter‐fractional variations. When selecting the appropriate FM for lung SBRT, a distance of 26 mm or less between the lung tumor and the FM can be one of the distance criteria.

## AUTHOR CONTRIBUTIONS

Yuki Manabe and Takehiro Shiinoki designed this study. All authors contributed to the interpretation of data and revision of the manuscript. Yuki Manabe wrote the manuscript with the support of Takehiro Shiinoki and Hidekazu Tanaka. All authors approved the final report for publication and agreed to be accountable for all aspects of this work.

## CONFLICT OF INTEREST STATEMENT

The authors have no conflicts of interest.

## References

[1] Tateishi Y , Takeda A , Horita N , et al. Stereotactic body radiation therapy with a high maximum dose improves local control, cancer‐specific death, and overall survival in peripheral early‐stage non‐small cell lung cancer. Int J Radiat Oncol Biol Phys. 2021;111(1):143‐151. doi:10.1016/j.ijrobp.2021.04.014 33891980

[2] Oliver DE , Laborde JM , Singh DP , et al. Early‐stage primary lung neuroendocrine tumors treated with stereotactic body radiation therapy: a multi‐institution experience [published online ahead of print, 2023 Jan 26]. Int J Radiat Oncol Biol Phys. 2023;S0360‐3016(23):00071‐00078. doi:10.1016/j.ijrobp.2023.01.028 PMC10845843 36708788

[3] Nagata Y , Hiraoka M , Shibata T , et al. Prospective trial of stereotactic body radiation therapy for both operable and inoperable T1N0M0 non‐small cell lung cancer: japan Clinical Oncology Group Study JCOG0403. Int J Radiat Oncol Biol Phys. 2015;93(5):989‐996. doi:10.1016/j.ijrobp.2015.07.2278 26581137

[4] Nguyen EK , Poon I , Ung YC , et al. Toxicity and efficacy of multitarget thoracic stereotactic body radiation therapy. Int J Radiat Oncol Biol Phys. 2023;115(4):897‐905. doi:10.1016/j.ijrobp.2022.10.032 36368432

[5] Helou J , Thibault I , Poon I , et al. Stereotactic ablative radiation therapy for pulmonary metastases: histology, dose, and indication matter. Int J Radiat Oncol Biol Phys. 2017;98(2):419‐427. doi:10.1016/j.ijrobp.2017.02.093 28463162

[6] Navarria P , Baldaccini D , Clerici E , et al. Stereotactic body radiation therapy for lung metastases from sarcoma in oligometastatic patients: a phase 2 study. Int J Radiat Oncol Biol Phys. 2022;114(4):762‐770. doi:10.1016/j.ijrobp.2022.08.028 35987453

[7] Erridge SC , Seppenwoolde Y , Muller SH , et al. Portal imaging to assess set‐up errors, tumor motion and tumor shrinkage during conformal radiotherapy of non‐small cell lung cancer. Radiother Oncol. 2003;66(1):75‐85. doi:10.1016/s0167-8140(02)00287-6 12559524

[8] Matsuo Y , Shibuya K , Nakamura M , et al. Dose–volume metrics associated with radiation pneumonitis after stereotactic body radiation therapy for lung cancer. Int J Radiat Oncol Biol Phys. 2012;83(4):e545‐e549. doi:10.1016/j.ijrobp.2012.01.018 22436782

[9] Guckenberger M , Baier K , Polat B , et al. Dose‐response relationship for radiation‐induced pneumonitis after pulmonary stereotactic body radiotherapy. Radiother Oncol. 2010;97(1):65‐70. doi:10.1016/j.radonc.2010.04.027 20605245

[10] Liu Y , Wang W , Shiue K , et al. Risk factors for symptomatic radiation pneumonitis after stereotactic body radiation therapy (SBRT) in patients with non‐small cell lung cancer. Radiother Oncol. 2021;156:231‐238. doi:10.1016/j.radonc.2020.10.015 33096168

[11] Meyers SM , Kisling K , Atwood TF , Ray X . A standardized workflow for respiratory‐gated motion management decision‐making. J Appl Clin Med Phys. 2022;23(8):e13705. doi:10.1002/acm2.13705 35737295 PMC9359043

[12] Mørkeset ST , Lervåg C , Lund JÅ , Jensen C . Clinical experience of volumetric‐modulated flattening filter free stereotactic body radiation therapy of lesions in the lung with deep inspiration breath‐hold. J Appl Clin Med Phys. 2022;23(9):e13733. doi:10.1002/acm2.13733 35867387 PMC9512343

[13] Ehrbar S , Perrin R , Peroni M , et al. Respiratory motion‐management in stereotactic body radiation therapy for lung cancer—A dosimetric comparison in an anthropomorphic lung phantom (LuCa). Radiother Oncol. 2016;121(2):328‐334. doi:10.1016/j.radonc.2016.10.011 27817945

[14] Anastasi G , Bertholet J , Poulsen P , et al. Patterns of practice for adaptive and real‐time radiation therapy (POP‐ART RT) part I: intra‐fraction breathing motion management. Radiother Oncol. 2020;153:79‐87. doi:10.1016/j.radonc.2020.06.018 32585236 PMC7758783

[15] Harada T , Shirato H , Ogura S , et al. Real‐time tumor‐tracking radiation therapy for lung carcinoma by the aid of insertion of a gold marker using bronchofiberscopy. Cancer. 2002;95(8):1720‐1727. doi:10.1002/cncr.10856 12365020

[16] Imura M , Yamazaki K , Shirato H , et al. Insertion and fixation of fiducial markers for setup and tracking of lung tumors in radiotherapy. Int J Radiat Oncol Biol Phys. 2005;63(5):1442‐1447. doi:10.1016/j.ijrobp.2005.04.024 16109463

[17] Shiinoki T , Kawamura S , Uehara T , et al. “Evaluation of a combined respiratory‐gating system comprising the TrueBeam linear accelerator and a new real‐time tumor‐tracking radiotherapy system: a preliminary study” [JACMP, 17(4), 2016]. J Appl Clin Med Phys. 2017;18(4):238. doi:10.1002/acm2.12125 28681447 PMC5874949

[18] Shiinoki T , Hanazawa H , Yuasa Y , Fujimoto K , Uehara T , Shibuya K . Verification of respiratory‐gated radiotherapy with new real‐time tumour‐tracking radiotherapy system using cine EPID images and a log file. Phys Med Biol. 2017;62(4):1585‐1599. doi:10.1088/1361-6560/aa587d 28072584

[19] Seppenwoolde Y , Shirato H , Kitamura K , et al. Precise and real‐time measurement of 3D tumor motion in lung due to breathing and heartbeat, measured during radiotherapy. Int J Radiat Oncol Biol Phys. 2002;53(4):822‐834. doi:10.1016/s0360-3016(02)02803-1 12095547

[20] Nakamura M , Takamiya M , Akimoto M , et al. Target localization errors from fiducial markers implanted around a lung tumor for dynamic tumor tracking. Phys Med. 2015;31(8):934‐941. doi:10.1016/j.ejmp.2015.06.012 26165177

[21] van der Voort van Zyp NC , Hoogeman MS , van de Water S , et al. Stability of markers used for real‐time tumor tracking after percutaneous intrapulmonary placement. Int J Radiat Oncol Biol Phys. 2011;81(3):e75‐e81. doi:10.1016/j.ijrobp.2010.12.026 21349655

[22] Casutt A , Kinj R , Ozsahin EM , von Garnier C , Lovis A . Fiducial markers for stereotactic lung radiation therapy: review of the transthoracic, endovascular and endobronchial approaches. Eur Respir Rev. 2022;31(163):210149. Published 2022 Jan 12. doi:10.1183/16000617.0149-2021 35022258 PMC9488911

[23] Ueki N , Matsuo Y , Nakamura M , et al. Intra‐ and interfractional variations in geometric arrangement between lung tumours and implanted markers. Radiother Oncol. 2014;110(3):523‐528. doi:10.1016/j.radonc.2014.01.014 24560763

[24] Roman NO , Shepherd W , Mukhopadhyay N , Hugo GD , Weiss E . Interfractional positional variability of fiducial markers and primary tumors in locally advanced non‐small‐cell lung cancer during audiovisual biofeedback radiotherapy. Int J Radiat Oncol Biol Phys. 2012;83(5):1566‐1572. doi:10.1016/j.ijrobp.2011.10.051 22391105

[25] Akasaka H , Mizonobe K , Oki Y , et al. Fiducial marker position affects target volume in stereotactic lung irradiation. J Appl Clin Med Phys. 2022;23(6):e13596. doi:10.1002/acm2.13596 35377962 PMC9195037

[26] Bhagat N , Fidelman N , Durack JC , et al. Complications associated with the percutaneous insertion of fiducial markers in the thorax. Cardiovasc Intervent Radiol. 2010;33(6):1186‐1191. doi:10.1007/s00270-010-9949-0 20661565 PMC2977074

[27] Kupelian PA , Forbes A , Willoughby TR , et al. Implantation and stability of metallic fiducials within pulmonary lesions. Int J Radiat Oncol Biol Phys. 2007;69(3):777‐785. doi:10.1016/j.ijrobp.2007.03.040 17606334

[28] Schroeder C , Hejal R , Linden PA . Coil spring fiducial markers placed safely using navigation bronchoscopy in inoperable patients allows accurate delivery of CyberKnife stereotactic radiosurgery. J Thorac Cardiovasc Surg. 2010;140(5):1137‐1142. doi:10.1016/j.jtcvs.2010.07.085 20850809

[29] Willmann J , Sidiqi B , Wang C , et al. Four‐dimensional computed tomography‐based correlation of respiratory motion of lung tumors with implanted fiducials and an external surrogate. Adv Radiat Oncol. 2021;7(3):100885. Published 2021 Dec 29. doi:10.1016/j.adro.2021.100885 35198837 PMC8792087

[30] Yamazaki R , Nishioka S , Date H , Shirato H , Koike T , Nishioka T . Investigation of the change in marker geometry during respiration motion: a preliminary study for dynamic‐multi‐leaf real‐time tumor tracking. Radiat Oncol. 2012;7:218. Published 2012 Dec 18. doi:10.1186/1748-717X-7-218 23249681 PMC3552716

[31] Smith RL , Yang D , Lee A , Mayse ML , Low DA , Parikh PJ . The correlation of tissue motion within the lung: implications on fiducial based treatments. Med Phys. 2011;38(11):5992‐5997. doi:10.1118/1.3643028 22047363 PMC3298561

[32] Nelson C , Balter P , Morice RC , et al. Evaluation of tumor position and PTV margins using image guidance and respiratory gating. Int J Radiat Oncol Biol Phys. 2010;76(5):1578‐1585. doi:10.1016/j.ijrobp.2009.08.002 20137865

[33] Yuasa Y , Shiinoki T , Onizuka R , Fujimoto K . Estimation of effective imaging dose and excess absolute risk of secondary cancer incidence for four‐dimensional cone‐beam computed tomography acquisition. J Appl Clin Med Phys. 2019;20(11):57‐68. doi:10.1002/acm2.12741 31593377 PMC6839364

